# A compare between myocardial topical negative pressure levels of -25 mmHg and -50 mmHg in a porcine model

**DOI:** 10.1186/1471-2261-8-14

**Published:** 2008-06-22

**Authors:** Sandra Lindstedt, Per Paulsson, Arash Mokhtari, Bodil Gesslein, Joanna Hlebowicz, Malin Malmsjö, Richard Ingemansson

**Affiliations:** 1 Department of Cardiothoracic Surgery, Lund University Hospital, Lund, Sweden; 2 Department of Medicine, Lund University Hospital, Lund, Sweden; 3 Department of Medicine, Malmö University Hospital, Malmö, Sweden

## Abstract

**Background:**

Topical negative pressure (TNP), widely used in wound therapy, is known to stimulate wound edge blood flow, granulation tissue formation, angiogenesis, and revascularization. We have previously shown that application of a TNP of -50 mmHg to the myocardium significantly increases microvascular blood flow in the underlying tissue. We have also shown that a myocardial TNP levels between -75 mmHg and -150 mmHg do not induce microvascular blood flow changes in the underlying myocardium. The present study was designed to elucidate the difference between -25 mmHg and -50 mmHg TNP on microvascular flow in normal and ischemic myocardium.

**Methods:**

Six pigs underwent median sternotomy. The microvascular blood flow in the myocardium was recorded before and after the application of TNP using laser Doppler flowmetry. Analyses were performed before left anterior descending artery (LAD) occlusion (normal myocardium), and after 20 minutes of LAD occlusion (ischemic myocardium).

**Results:**

A TNP of -25 mmHg significantly increased microvascular blood flow in both normal (from 263.3 ± 62.8 PU before, to 380.0 ± 80.6 PU after TNP application, * *p *= 0.03) and ischemic myocardium (from 58.8 ± 17.7 PU before, to 85.8 ± 20.9 PU after TNP application, * *p *= 0.04). A TNP of -50 mmHg also significantly increased microvascular blood flow in both normal (from 174.2 ± 20.8 PU before, to 240.0 ± 34.4 PU after TNP application, * *p *= 0.02) and ischemic myocardium (from 44.5 ± 14.0 PU before, to 106.2 ± 26.6 PU after TNP application, ** *p *= 0.01).

**Conclusion:**

Topical negative pressure of -25 mmHg and -50 mmHg both induced a significant increase in microvascular blood flow in normal and in ischemic myocardium. The increase in microvascular blood flow was larger when using -25 mmHg on normal myocardium, and was larger when using -50 mmHg on ischemic myocardium; however these differences were not statistically significant.

## Background

TNP promotes wound healing by stimulating wound edge blood flow, as has been shown in both peripheral [1] and in skeletal muscle in sternotomy wounds[2]. TNP produces a mechanical stress and a pressure gradient across the tissue which may cause a surge of blood to the area. Mechanical forces and increased blood flow are known to stimulate granulation tissue formation, i.e. endothelial proliferation, capillary budding and angiogenesis[3,4].

Poststernotomy mediastinitis is a strong predictor for poor long-term survival after coronary artery by-pass surgery (CABG), when using conventional wound healing techniques (closed irrigation, delayed wound closure, or reconstructing with omentum or pectoral flaps)[5,6]. Braxton and coworkers demonstrated that actuarial survival after 10 years was 39% in patients with poststernotomy mediastinitis and 70% in patients without mediastinitis[6]. Milano and collages have suggested that mediastinitis may cause negative long-terms effects on several organs such as the heart and kidneys[5]. Lately, the usefulness of TNP in the treatment of poststernotomy mediastinitis has been well recognized among cardiothoracic surgeons around the world, due to excellent clinical outcome[7,8].

Previously, we have showed no difference in long-term survival between CABG patients with TNP-treated mediastinitis and CABG patients without mediastinitis[9]. This might indicate that these patients have developed increased coronary collateral blood vessels during TNP, since the anterior part of the heart is in direct contact with the negative pressure source during TNP treatment, and they might therefore be better prepared when bypass grafts fail to work. It may be that the TNP stimulation of blood flow and development of collateral blood vessels in part account for the reduced long-term mortality in those patients.

Earlier, we have showed that a TNP of -50 mmHg significantly increases microvascular blood flow in normal, ischemic, and reperfused porcine myocardium[10]. We have also demonstrated that TNP levels between -75 mmHg and -150 mmHg do not induce microvascular blood flow changes in normal nor ischemic porcine myocardium[11]. When applying TNP to subcutaneous tissue and skeletal muscle, a relative zone of hypoperfusion is seen close to the vacuum source. The size of the zone depends on tissue density, the amount of negative pressure applied, and the distance from the vacuum source[12]. We believe that application of a TNP of -150 mmHg results in hypoperfusion 6–8 mm down into the ischemic myocardium, which would explain previous findings[11]. A large zone of hypoperfusion in the myocardium would, theoretically lead to ischemia in those parts of the myocardium, and would, potentially cause damage to the heart.

The aim of this study was thus to elucidate the differences between negative pressures of -25 mmHg and -50 mmHg. Microvascular blood flow was measured in a porcine model using laser Doppler flowmetry. The effects of TNP of -25 mmHg and -50 mmHg on microvascular blood flow were investigated in the myocardium before (normal myocardium) and during occlusion of the left anterior descending artery (LAD) (ischemic myocardium) to imitate an ischemic coronary artery disease.

## Methods

### Experimental animals

A porcine model was use for the present study. Six domestic landrace pigs of both genders, with a mean body weight of 70 kg, were fasted overnight with free access to water. The study was approved by the Ethics Committee for Animal Research, Lund University, Sweden. The investigation complied with the "Guide for the Care and Use of Laboratory Animals" as recommended by the U.S. National Institutes of Health, and published by the National Academies Press (1996).

### Anesthesia

All the animals were pre-medicated intramuscularly with ketamine (30 mg/kg) before they were brought into the laboratory. Before commencing surgery, sodium thiopental (5 mg/kg), atropine (0.02 mg/kg) and pancuronium (0.5 mg/kg) were given intravenously. Tracheotomy was performed with a Portex endo-tracheal tube (7.5 mm internal diameter, Medcompare™, USA). A servo-ventilator (Siemens Elema 300A, Stockholm, Sweden) was used for mechanical ventilation throughout the experiment. The ventilator settings used were: minute volume = 100 ml/kg, FiO_2 _= 0.5, breathing frequency = 16 breaths/minute and positive end expiratory pressure = 5 cmH_2_O.

Anesthesia and muscular paralysis were maintained by continuous intravenous infusion of Diprivan^® ^(propofol, AstraZeneca, Sweden) 8–10 mg/kg/hour, Leptanal^® ^0.15 mg/kg/hour (fentanyl, Lilly, France) and Pavulon^® ^0.6 mg/kg/hour (pancuronium, Organon Teknika, Boxtel, the Netherlands).

### Data acquisition

Mean arterial pressure, central venous pressure, body temperature, and ventilator parameters were recorded throughout the experiments.

### Surgical procedure

Surgery was performed through median sternotomy. After heparinization (400 IU/kg) a cardiopulmonary bypass (CPB) was installed with an arterial cannula (22 French, DLP^® ^Elongated One-Piece Arterial Cannula (EOPA™), Medtronic Inc., Minneapolis, MO, USA) in the distal ascending aorta, and a venous cannula (32 French, MC2^® ^Two-Stage Venous Cannula, Medtronic Inc.) inserted through the right atrium. Before cannulation of the heart the cannulae were inserted through the thoracic wall to prevent air leakage during TNP application. CPB was conducted in normothermia. Ventricular fibrillation was subsequently induced in the heart. No aortic cross-clamping was performed and no cardioplegia was employed. The mean arterial pressure was maintained between 60 and 80 mmHg. A left ventricular vent (DLP^® ^Vent, Medtronic Inc.) was used to protect the left chamber from overloading. Pulmonary ventilation was applied at 4 liters/minute during the experiments.

CPB was used to facilitate the measurement of microvascular blood flow using laser Doppler flowmetry. Fibrillation of the heart minimizes the movement artifacts, while the physiological conditions are, to a large extent, conserved. Moreover, CPB prevents the risk of circulatory failure during LAD occlusion, thereby facilitating experimental analysis in the ischemic myocardium.

Microvascular blood flow was measured by laser Doppler flowmetry (Peri Flux System 5000, Perimed, Stockholm, Sweden), using a technique that quantifies the sum of the motion of the red blood cells in a specific volume, extensively applied in plastic surgery procedures[13]. In this method, a fiber optic probe carries a beam of light. Light impinging on cells in motion undergoes a change in wavelength (Doppler shift) while light impinging on static objects remains unchanged. The magnitude and frequency distribution of the changes are directly related to the number and velocity of red blood cells. The information is collected by a returning fiber, converted into an electronic signal and analyzed.

Laser Doppler probes were inserted horizontally into the heart muscle 6–8 mm lateral of the LAD at depths of approximately, 6–8 mm. The mean thickness of the chamber wall was approximately 20–25 mm. All probes were carefully fixed to the surface of the heart with a suture (Prolene 7-0; Ethicon Inc., Somerville, NJ, USA), thereby preventing the probe from sliding. A round hole with a diameter of 5 cm was made in the middle of a Phrenic Nerve Pad^®^(Medtronic Inc.) and placed on top of the heart. The pad was stabilized to the surrounding myocardium with 8–10 sutures (Prolene 5-0; Ethicon Inc.) and to the posterior sternal edges with sutures (Dermalon 2-0; Davis and Geck, St Louis, MO, USA). A retractor was used throughout the experiments to keep the sternal edges apart. A polyurethane foam dressing, with an open pore structure of 400 to 600 μm (KCI, Copenhagen, Denmark) was placed between the sternal edges. The foam was continuously sutured to the surrounding skin (Dermalon 2-0; Davis and Geck). The wound was then sealed with a transparent adhesive drape. A track pad (KCI) was inserted through the drape and was connected to a continuous vacuum source, (V.A.C. pump unit, KCI). When the negative pressure is applied, the heart will be drawn up towards the phrenic nerve pad and the foam without interfering with the sternal edges. This procedure causes the application of negative pressure to affect only the myocardium exposed by the 5 cm diameter hole. After the experiment the heart was dissected to confirm probe location.

### Experimental protocol

The microvascular blood flow was measured continuously by the laser Doppler filament probes. Recordings were made in normal myocardium before and after negative pressures of -25 or -50 mmHg were applied. In animal 1, 3, and 5 the negative pressure of -25 mmHg was applied before the negative pressure of -50 mmHg was applied. The baseline was restored between the two settings. In animal 2, 4, and 6 the negative pressure of -50 mmHg was applied before the negative pressure of -25 mmHg was applied. The baseline was restored between the two settings.

The LAD was then occluded for 20 minutes with an elastic vessel loop. Microvascular blood flow was measured before, and after 20 minutes of occlusion.

Recordings were also made in ischemic myocardium, before and after negative pressures of -25 or -50 mmHg were applied. In animal 1, 3, and 5 the negative pressure of -25 mmHg was applied before the negative pressure of -50 mmHg was applied. The baseline was restored between the two settings. In animal 2, 4, and 6 the negative pressure of -50 mmHg was applied before the negative pressure of -25 mmHg was applied. The baseline was restored between the two settings.

### Calculation and statistics

Laser Doppler flowmetry measurements were performed on six pigs. The output was continuously recorded using PeriSoft software (Perimed, Stockholm, Sweden). Microvascular blood flow was expressed in terms of perfusion units (PU). Calculations and statistical analysis were performed using GraphPad 4.0 software. Statistical analysis was performed using Student's paired t-test, and significance was defined as ***p < 0.001, **p < 0.01, *p < 0.05, and *p *> 0.05 (not significant, n.s.). Values are presented as means ± the standard error on the mean (SEM).

## Results

### Normal myocardium

**Figure 1:**
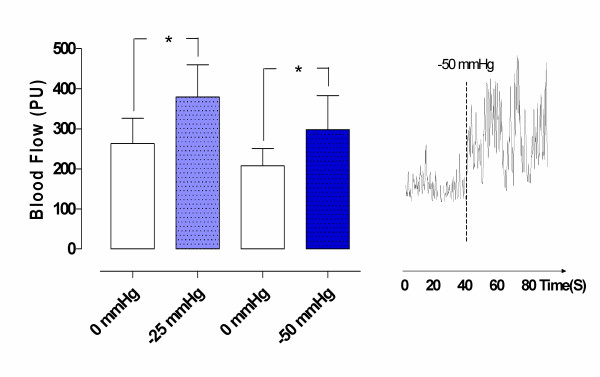
**Microvascular blood flow measured using laser Doppler flowmetry in normal myocardium exposed to topical negative pressures of -25 mmHg and -50 mmHg.** The measurements were performed at a depth of 6–8 mm in the myocardium in six pigs. The results are shown as mean values ± SEM in the left panel. A level of *p < 0.05, **p < 0.01, and ***p < 0.001 was considered statistically significant. The right panel shows a representative example of microvascular blood flow changes before and after application of -50 mmHg. Note the immediate blood flow response when negative pressure is applied.

A topical negative pressure of -25 mmHg significantly increased microvascular blood flow in normal myocardium (from 263.3 ± 62.8 PU before, to 380.0 ± 80.6 PU after TNP application, * *p *= 0.03). A TNP of -50 mmHg also significantly increased microvascular blood flow in normal myocardium (from 174.2 ± 20.8 PU before, to 240.0 ± 34.4 PU after TNP application, * *p *= 0.04) (Figure 1).

### LAD occlusion

**Figure 2:**
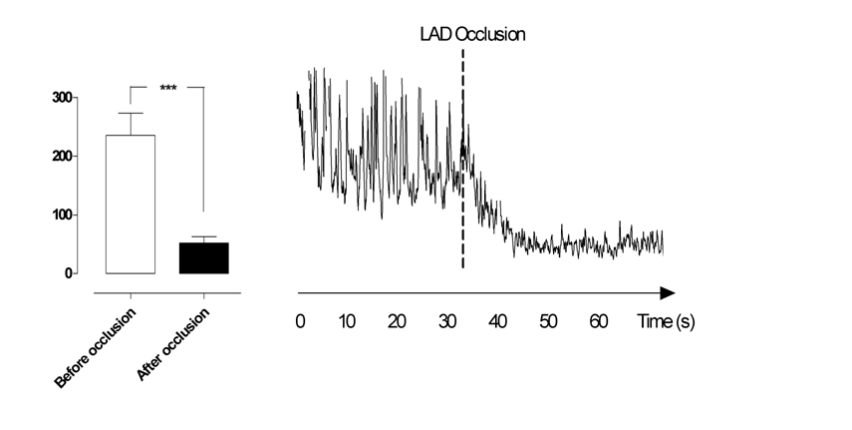
**Microvascular blood flow measured using laser Doppler flowmetry in the myocardium before and after 20 minutes of occlusion of the left anterior descending artery (LAD).** The measurements were performed at a depth of 6–8 mm in the myocardium in six pigs. The results are shown as mean values ± SEM in the left panel. A level of *p < 0.05, **p < 0.01, and ***p < 0.001 was considered statistically significant. The right panel shows a representative example of microvascular blood flow changes before and after occlusion of the LAD

Ischemia was induced by occlusion of the LAD for 20 minutes. The blood flow was 235.4 ± 37.4 PU before occlusion of the LAD and decreased to 52.1 ± 11.1 PU after 20 minutes of LAD occlusion (*** *p *= 0.003) (Figure 2).

### Ischemic myocardium

**Figure 3:**
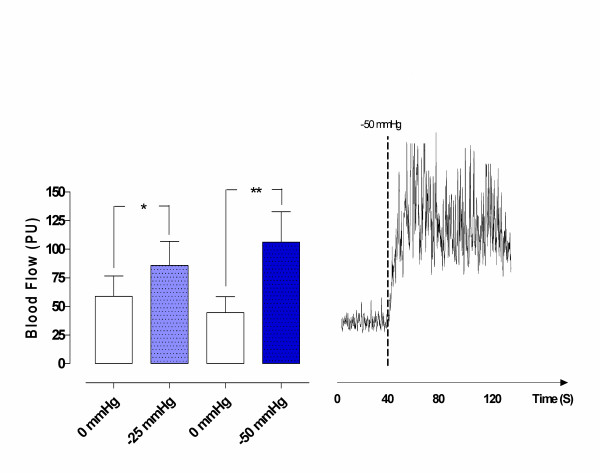
**Microvascular blood flow measured using laser Doppler flowmetry in ischemic myocardium, after 20 minutes of occlusion of the left anterior descending artery, exposed to topical negative pressures of -25 mmHg and -50 mmHg.** The measurements were performed at a depth of 6–8 mm in the myocardium in six pigs. The results are shown as mean values ± SEM in the left panel. A level of *p < 0.05, **p < 0.01, and ***p < 0.001 was considered statistically significant. The right panel shows a representative example of microvascular blood flow changes before and after application of -50 mmHg. Note the immediate blood flow response when negative pressure is applied.

A topical negative pressure of -25 mmHg significantly increased microvascular blood flow in ischemic myocardium (from 58.8 ± 17.7 PU before, to 85.8 ± 20.9 PU after TNP application, * *p *= 0.02). A TNP of -50 mmHg also significantly increased microvascular blood flow in ischemic myocardium (from 44.5 ± 14.0 PU before, to 106.2 ± 26.6 PU after TNP application, ** *p *= 0.01) (Figure 3).

## Discussion

Poststernotomy mediastinitis is a strong predictor for poor long-term survival after coronary artery by-pass grafting (CABG) [5,6]. Braxton and coworkers demonstrated that actuarial survival after 10 years was 39% in patients with poststernotomy mediastinitis and 70% in patients without mediastinitis[6]. Milano and collages have suggested that mediastinitis may cause negative long-terms effects on several organs such as the heart and kidneys[5]. Theoretically, a massive immunological response during a prolonged period of infection may cause adverse effects on by-pass grafts. In those studies, reporting poor long-term survival after mediastinitis, several conventional wound healing techniques were used (closed irrigation, delayed wound closure, or reconstructing with omentum or pectoral flaps). Interestingly, Sjogren and coworkers, found no difference in long-term survival between CABG patients with TNP-treated mediastinitis and CABG patients without mediastinitis [9]. During the treatment of poststernotomy mediastinitis, the topical negative pressure is in direct contact with the heart, which is exposed through the diastase of the sternotomy. These patients may therefore have developed increased coronary collateral blood vessels during TNP, and may be better prepared when bypass grafts fail to work. We have indeed observed in patients treated with TNP, that richly vascularized granulation tissue develops over the heart within 8–10 days. It may well be that the stimulation of blood flow and the development of collateral blood vessels resulting from TNP in part account for the reduced long-term mortality in patients treated in this way.

TNP has become the therapy of choice in the treatment of chronic and problematic wounds at many hospitals worldwide, due to excellent clinical outcome[9,14]. Despite the extensive clinical use and excellent outcome of TNP in wound therapy, the fundamental scientific mechanism is only partly understood. The known effects of TNP are enhanced blood flow to the wound edge and granulation tissue formation [15,16]. TNP increases blood flow velocity and opens up the capillary beds. The mechanical force (vacuum force) exerted by TNP and increased blood flow and shear stress against the vessel walls, which affect the cytoskeleton in the vascular cells, stimulating endothelial proliferation and angiogenesis[3,17]. TNP has recently been shown to increase angiogenesis and decrease matrix metalloproteinases both of which promote new vessel formation [4].

In a previous report we applied a TNP of -50 mmHg over the LAD region on six pigs, and was able to show a significant increase in microvascular blood flow in normal myocardium (non-ischemic myocardium), in ischemic myocardium after 20 minutes of LAD occlusion, and also in reperfused myocardium (former ischemic myocardium after 20 minutes of reperfusion)[10]. In another previous report we show that pressure levels between -75 and -150 mmHg did not induce any significant microvascular blood flow changes in normal neither ischemic myocardium[11]. When applying negative pressure to subcutaneous tissue and skeletal muscle, a relative zone of hypoperfusion is seen close to the vacuum source. The size of the hypoperfusion zone depends on tissue density, the amount of negative pressure applied, and the distance from the vacuum source[12]. We believe that pressure levels above -75 mmHg results in vasoconstriction 6–8 mm down into the underlying myocardium, which would explain previous findings[11]. A large zone of vasoconstriction in the myocardium would, theoretically lead to ischemia in those parts of the myocardium, and would, potentially cause damage to the heart. Theoretically, the most beneficial topical negative pressure, to use on the myocardium, should be as low as possible, to avoid a zone of vasoconstriction. Moreover, the negative pressure has to be high enough to create a vacuum force big enough to cause a significant increase in the microvascular blood flow in the underlying myocardium. In the present study we show that a myocardial topical negative pressure of -25 mmHg results in an increase in microvascular blood flow in the underlying tissue in both normal and ischemic myocardium. We also show that a myocardial topical negative pressure of -50 mmHg results in a microvascular blood flow increase (normal and ischemic myocardium), as shown in prior study[11]. Moreover, the increase in microvascular blood flow was larger when using a TNP of -25 mmHg on normal myocardium, and was larger when using a TNP of -50 mmHg on ischemic myocardium, however these differences were not statistically significant, and might therefore be analyzed with caution. From our perspective a topical negative pressure of -50 mmHg might be more favourable as optimal myocardial TNP than -25 mmHg.

In the present study, CPB was used, which facilitated the intervention since arrhythmia and circulatory failure were avoided during the induction of ischemia. Laser Doppler flowmetry is a well established method that provides continuous recording of the microvascular blood flow. To use laser Doppler on a beating heart is complex. Every heart beat provides a movement that will become registered by the laser Doppler flowmetry[18]. The movement artifact of the laser Doppler flow curve becomes very large and the data collected becomes difficult to analyze. The experiment was conducted during low intense ventricular fibrillation to minimize movement artifacts from heart beats, and minimize tissue trauma, and sliding of the laser Doppler probes, while measuring blood flow in the myocardium[18]. Moreover, ventricular fibrillation does differ from sinus rhythm. During ventricular fibrillation there is no synchronized contraction in the ventricles. If perfusion pressure is kept above 50 mmHg the ventricular wall will still be perfused[19], but the myocardial wall tension may become higher than in a beating heart[20]. Hottenrot et al. showed in 1972 that perfusion of a fibrillating dog heart for one to two hours did not damage the subendocardial muscle unless a strong maintained electrical stimulus was used[21]. In the present study, no sustained electrical stimuli was used, the ventricle was protected from distension with a left ventricular vent, and the perfusion pressure was be kept at 80 mmHg, to avoid subendocardial ischemia in the fibrillating heart [22]. The effect of TNP on the beating heart can not be deduced from the present results, although we believe that the effect would be similar to that observed here.

The future clinical application of myocardial TNP is still too early to determine. However, the potential application of myocardial TNP speculatively, in the future, might become in the treatment of patients with extensive coronary disease or/and refractory angina pectoris but also in the acute phase of a ST-elevation myocardial infarction. No-reflow sometimes occurs when using percutaneous coronary intervention (PCI) to treat cases of acute ST-elevation myocardial infarction. The treatment of established no-reflow is mainly pharmacological, since the obstruction occurs at the microvasculature level. Compared with patients in whom no-reflow is transient, refractory no-reflow is associated with a markedly increased risk of 30-day mortality[23,24]. To apply TNP at those areas in the acute phase, with for example a trans-thoracic device, might be useful for those patients. The method might also, in the future, serve as an alternative treatment for patients with extensive coronary artery disease, since, VEGF proteins, that are also stimulated by TNP[25,26], have been shown to play a key role in the modulation of angiogenesis and vascular growth [27]. A transthoracic device without CPB would be needed. Patients with refractory angina pectoris represent a clinical challenge due to the absence of a proven effective treatment. Many different therapies have been tried, such as percutaneous myocardial laser revascularization[28], and enhanced external counter-pulsation[29], with varying results. Therapeutic angiogenesis, wherein exogenous growth factors are administered to ischemic tissue to enhance collateral vessel formation and reperfusion, have also been investigated as a potential form of treatment for these patients. However, despite promising laboratory results, clinical studies have not shown any clear benefit. Vascular endothelial growth factor (VEGF) proteins have been shown to play a key role in the modulation of angiogenesis and vascular growth[27]. Interestingly, TNP produces a mechanical shear stress that is known to activate endogenous VEGF[25,26].

## Conclusion

In conclusion, TNP of -25 mmHg and -50 mmHg both induce a significant increase in microvascular blood flow in normal and in ischemic myocardium. Moreover, the increase in microvascular blood flow was larger when using a TNP of -25 mmHg on normal myocardium, and was larger when using a TNP of -50 mmHg on ischemic myocardium, however these differences were not statistically significant, and might therefore be analyzed with caution. From our perspective a topical negative pressure of -50 mmHg might be more favourable as optimal myocardial TNP than -25 mmHg.

## Abbreviations

CABG: Coronary Artery Bypass Grafting; LAD: Left Anterior Descending Artery; PCI: Percutaneous Coronary Intervention; PU: Perfusion Units; TNP: Topical Negative Pressure; VEGF: Vascular Endothelial Growth Factor.

## Competing interests

The authors declare that they have no competing interests.

## Authors' contributions

SL developed, conceived, carried out, and coordinated the animal studies, analyzed data, drafted and wrote the manuscript. MM participated in the animal experiments and gave valuable analysis of the manuscript. RI developed the animal model, and participated in the animal experiments, and also gave valuable advice on both animal experiments and the manuscript. BG participated in the animal experiments. PP and AM gave valuable analysis of the manuscript. JH gave valuable statistical analysis of the manuscript. All authors read and approved the final manuscript.

## Pre-publication history

The pre-publication history for this paper can be accessed here:


